# Longitudinal evaluation of dehydroepiandrosterone (DHEA), its sulfated form and estradiol with cancer-related cognitive impairment in early-stage breast cancer patients receiving chemotherapy

**DOI:** 10.1038/s41598-022-20420-3

**Published:** 2022-10-03

**Authors:** Yi Long Toh, Chia Jie Tan, Ning Yi Yap, Ritesh Parajuli, Aik Jiang Lau, Alexandre Chan

**Affiliations:** 1 Department of Pharmacy, Faculty of Science, National University of Singapore, Singapore, Singapore; 2 Department of Medicine, University of California, Irvine, USA; 3 Department of Pharmacology, Yong Loo Lin School of Medicine, National University of Singapore, Singapore, Singapore; 4 College of Pharmacy, Dalhousie University, Halifax, Canada; 5 Department of Pharmacy, National Cancer Centre Singapore, Singapore, Singapore; 6 Department of Clinical Pharmacy Practice, University of California Irvine, Irvine, CA USA

**Keywords:** Biomarkers, Oncology

## Abstract

The purpose of this study is to elucidate how patient-reported cognitive symptoms manifest from variations in hormone levels or precursors such as dehydroepiandrosterone (DHEA) and its sulfated form [collectively termed as DHEA(S)] and to investigate their association in breast cancer survivors. Levels of estradiol and DHEA(S) were compared between early-stage breast cancer patients with and without cancer-related cognitive impairment (CRCI) during adjuvant chemotherapy. Data were analyzed from 242 patients (mean age ± SD = 50.8 ± 9.2 years) who had completed FACT-Cog v.3.0, blood draws and questionnaires. Regression model was used to fit the magnitude of change in each respective biomarker levels against overall cognitive impairment status while adjusting for clinically important covariates. There was reduction in mean plasma levels of estradiol and DHEAS during and towards the end of chemotherapy (*p*-values < 0.001). Compared to non-impaired patients, smaller magnitude of decline was observed in DHEA(S) levels in patients reporting CRCI, with significant association between decline in DHEAS levels and acute onset of CRCI at 6 weeks from baseline (adjusted β of 0.40, *p*-value of 0.02). In contrast, patients reporting CRCI showed greater magnitude of decline in estradiol compared to non-impaired patients, although this was not found to be statistically significant. There was an association between magnitude of change in biomarker levels with self-reported CRCI which suggests that the hormonal pathway related to DHEAS may be implicated in acute CRCI for breast cancer survivors. Our findings help to improve biological understanding of the pathway from which DHEAS may correlate with cognitive dysfunction and its impact on cancer survivors.

## Introduction

Cancer-related cognitive impairment (CRCI) is characterised by changes to cognitive domains of mainly memory, processing speed, attention and executive function, after receiving cancer treatment^1^. It is a problem widely reported in breast cancer patients receiving chemotherapy and its effects may persist through varying cognitive trajectories, causing a detrimental impact on quality of life even after completion of treatment^2^. While most clinical studies primarily focused on the effects of chemotherapy on cognition, cognitive deficits had been reported in patients receiving hormonal therapy and adjuvant endocrine therapy amongst other treatment modalities^3,4^. In the context of breast cancer, although endocrine therapy is known to induce cognitive deficits in cancer patient population^5–9^, few studies had explored how the variation in hormone levels either through suppression of hormone levels or reduction of precursors’ production may lead to the manifestation of patient-reported CRCI symptoms during adjuvant chemotherapy.

During chemotherapy, agents such as cyclophosphamide can cause ovarian insufficiency which may induce a transient menopausal state, leading to suppression of female hormones such as estradiol^10,11^. Suppression of estradiol may lead to interference with cognitive processes^12^. Postmenopausal women with higher circulating estradiol levels appeared less likely to suffer from cognitive impairment and had better memory performance, suggesting a protective role of estradiol in domains related to memory and verbal fluency^13–15^. Moreover, this effect is not found to be gender-specific as lower estradiol levels significantly correlated with worse cognitive scores^16^ and estradiol decline had been reported to be associated with cognitive domains of verbal fluency, visual recognition and visual memory in men with prostate carcinoma^17^. The observed effect of cognitive deficits being induced in patients receiving endocrine therapy and/or hormonal deprivation therapy suggest that suppression of hormone levels may be linked to cognitive impairment. An appreciation of estradiol’s role in the context of cognition may be central to understanding the influence of steroidal hormone synthesis pathway on CRCI.

**Figure 1:**
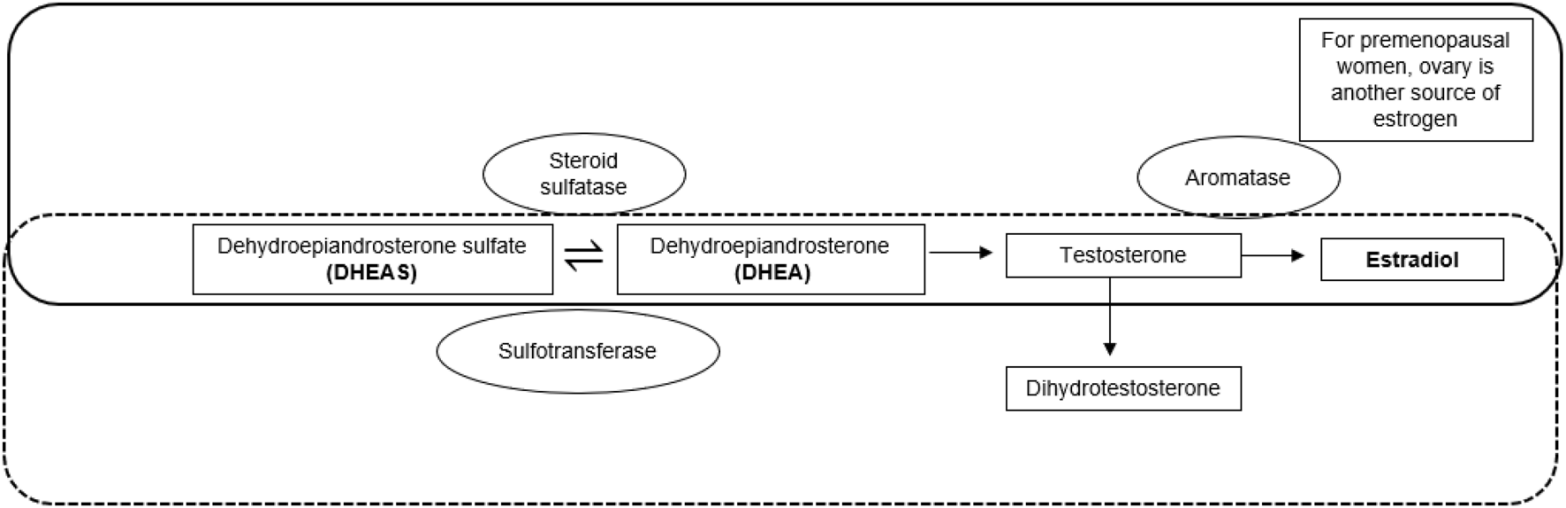
Schematic diagram showing the steroidogenesis pathway involving DHEA(S) and estradiol.

To further appreciate the quantitative relationship between hormone levels and cognitive performance, we measured estradiol levels relative to levels of dehydroepiandrosterone (DHEA) and its sulfated form [collectively termed as DHEA(S)]. As the precursors for the biosynthesis of steroidal hormones, DHEA(S) can be converted to androgens or estrogens in peripheral tissues (Fig. 1)^18^. Findings from our previous study had shown that higher pre-chemotherapy plasma levels of DHEAS were associated with lower odds of developing CRCI, in domains of verbal fluency and mental acuity^19^. In clinical studies, higher DHEAS levels were associated with better cognitive performance in healthy older adults^20–22^ while cancer survivors with higher DHEAS levels had reported better performance in attention domain^23^.

Taken together, we hypothesized that lower levels of DHEA(S) and estradiol would confer less neuroprotection and be associated with CRCI, given the role of these biomarkers in modulating cognitive function. In this study, our primary objective was to examine changes in DHEA(S) and estradiol levels between early-stage breast cancer patients with and without CRCI, during adjuvant chemotherapy. As menopausal status may obscure the observed effect associated with cognitive changes^24,25^, our secondary objective was to examine the impact of menopausal status on the variability in biomarker levels.

## Methods

### Study design

Data was obtained and analyzed from two prospective cohort studies conducted at the National Cancer Centre Singapore (NCCS), KK’s Women and Children Hospital (KKH) and Changi General Hospital (CGH) between 2011–2016 and 2014–2017. The studies had been approved by the SingHealth Institutional Review Board (CIRB 2011/457/B and 2014/754/B). Written informed consent was obtained from patients prior to their study participation. Research was performed in accordance with the Declaration of Helsinki and all methods (including experiments) were performed in accordance to relevant guidelines and regulations.

Patients who were scheduled to receive chemotherapy and fulfilled the following eligibility criteria were referred to the study team by their oncologists. The inclusion criteria were: (i) at least 21 years of age, (ii) diagnosed with early-stage breast cancer (stages I-III), (iii) scheduled to receive chemotherapy, (iv) had no prior exposure to chemotherapy and/or radiation therapy and (v) capable of providing informed consent. Patients who were diagnosed with neurocognitive disorders were excluded.

All participants completed study procedures at the following time points: before initiation of chemotherapy (baseline; T1), during chemotherapy which is approximately 6 weeks after baseline (T2) and at completion of chemotherapy which is approximately 12 weeks after baseline (T3). Relevant demographic and clinical data were clerked from patient interviews and electronic medical records. A 10-mL blood sample was drawn at each study time point and stored in ethylenediaminetetraacetic acid (EDTA) tube. The blood sample was centrifuged at 1069 × *g* for 10 min within 30 min of collection and the extracted plasma was stored at −80 °C until sample analysis.

### Study tools

At each study time point, a series of patient-reported outcome questionnaires were completed. The Brief Fatigue Inventory, Beck Anxiety Inventory and the European Organization for Research and Treatment of Cancer Quality of Life Questionnaire (EORTC QLQ-C30) were administered to capture patients’ fatigue levels, anxiety symptoms and health-related quality of life respectively. The symptom scale of the EORTC was used to assess for insomnia in our patient cohort. For evaluation of patient-reported cognitive function, the Functional Assessment of Cancer Therapy-Cognitive Function (FACT-Cog) Version 3.0 was used. It is a questionnaire used to assess the impact on patient’s quality of life within the past 7 days. The use of the English version had been previously validated within the Asian breast cancer population with good internal consistency across all cognitive domains (α = 0.70 to 0.93)^26^. Items in the subscale were mapped into sub-domains of mental acuity, concentration, memory, verbal fluency, functional interference and multitasking^27^. The status of overall cognitive impairment was defined as having a reduction of 10.6 points on the global FACT-Cog score between at least two assessed time points (T2 relative to T1 for 6 weeks; T3 relative to T1 for 12 weeks), based on an established minimal clinical important difference (MCID)^28^. A sensitivity analysis was also conducted using the Perceived Cognitive Impairment (PCI) sub-scale to classify the status of cognitive impairment^29^.

### Quantification of biomarker levels

Plasma was analysed for levels of estradiol using a commercially available enzyme-linked immunosorbent assay (ELISA) kit (Cayman Chemicals, Ann Arbor, Michigan). Standards, controls, and samples were added in duplicates to 96-well plates and analysed according to manufacturer’s recommendation. Plates were read based on absorbance values at 414 nm. Four parameter logistic regression was used to construct a standard curve, which was then used to calculate control and unknown concentrations of samples. The related precursor DHEA(S) levels were quantified using the liquid chromatography-mass spectrometry method previously outlined^19^, with negative electrospray ionization (ESI) mode to quantify DHEAS at mass/charge ratio of 367.0  97.0 and positive ESI to quantify for DHEA at mass/charge of 289.1  253.6, together with their respective internal standards. The obtained concentrations were then converted to standard molar units.

### Statistical analysis

Descriptive statistics were used to summarize demographic and clinical characteristics of patients. The number of patients with CRCI relative to baseline (T2-T1 or T3-T1) was described as proportions and compared between menopausal status using chi-square test. Repeated measures analysis of variance (ANOVA) was used to evaluate the change in plasma biomarker levels across time points, followed by post-hoc paired t-test to identify the time points in which change occurs. Bonferroni correction for multiple testing was applied for post hoc comparison. Regression model was fitted with magnitude of change in biomarker levels between study time points as the dependent variable and impairment status as an independent variable. The beta-coefficient (β) of the impairment status was considered as the estimated effect (as an estimated difference between group averages) and a separate model was fitted for each study timepoint relative to baseline. Each beta-coefficient represents the difference in mean levels, with a positive β representing greater extent in magnitude of change for the non-impaired group (reference group) compared to impaired group, after controlling for the covariates. Clinically relevant factors such as baseline fatigue, anxiety, insomnia which have been identified to be symptoms associated with worse perceived cognition as a cluster^30^, age^14^ and baseline menopausal status were chosen to be adjusted for. With reference to our secondary objective, baseline menopausal status was also considered for interaction with cognitive impairment status. All statistical analyses were performed with STATA version 16 (StataCorp, College Station, TX, 2017), and two-sided *p*-values less than 0.05 were considered statistically significant.

## Results

### Patient characteristics

There was a total of 242 participants (Supplementary Fig. 1); patients with missing blood samples or patient questionnaires at any study time point were excluded from analysis (Supplementary Table 1). The mean age ± standard deviation (SD) of participants was 50.8 ± 9.2 years and mean body mass index was 24.5 ± 4.3 kg/m^2^. Majority of the patients was diagnosed with stage II breast cancer (63.2%) and received anthracycline-based chemotherapy (68.6%), with other baseline characteristics summarised in Table 1. A total of 29.3% of patients reported self-perceived CRCI during the study period. For the individual sub-domains, the proportion of patients impaired in mental acuity (29.3%), concentration (26.1%), memory (15.8%), verbal fluency (18.2%), functional interference (18.7%) and multitasking (26.2%) were listed under Table 2. Upon stratifying patients by their menopausal status, there was a higher proportion of pre-menopausal patients reporting impairment in mental acuity (35.2% vs 23.3%, *p*-value (*p*) = 0.042) and concentration domain (35.0% vs 17.6%, *p* = 0.03).

**Table 1:** Baseline demographic and clinical characteristics of the patients.

Demographic information		Pooled Cohort^a^ (n = 242)
Age (years)		50.8 ± 9.2
Years of education		11.2 ± 3.4
Body mass index (kg/m^2^)		24.5 ± 4.3
Ethnicity	Chinese	203 (83.9)
	Malay	23 (9.5)
	Indian	9 (3.7)
	Others	7 (2.9)
Breast cancer stage	I	31 (12.8)
	II	153 (63.2)
	III	58 (24.0)
ECOG status	0	229 (94.6)
	1	13 (5.4)
Chemotherapy regimen	Anthracycline-based	166 (68.6)
	Taxane-based	76 (31.4)
Menopausal status	Pre-menopausal	122 (50.4)
	Post-menopausal	120 (49.6)
Estradiol (pM)		191.9 ± 232.2
DHEAS (µM)		2.8 ± 1.9
DHEA (nM)		14.5 ± 10.9
Baseline fatigue scores^b^		1.7 ± 1.8
Baseline anxiety scores^c^		6.8 ± 6.9
Baseline insomnia scores^d^		21.7 ± 26.1

^a^Data are presented as mean ± standard deviation for continuous variables and frequency (%) for categorical variables.

^b^Fatigue scores are out of maximum of 10 points, with higher score indicating greater extent of fatigue. ^c^Anxiety scores are out of maximum of 63 points, with higher score indicating greater extent of anxiety. ^d^Insomnia scores are out of maximum of 100 points, with higher score indicating greater extent of insomnia.

**Table 2:** Proportion of patients reporting self-perceived cancer-related cognitive impairment (CRCI).

Cognitive impairment based on:	Pooled Cohort^a^ (n = 242)	Pre-menopausal^a^ (n = 122)	Post-menopausal^a^ (n = 120)	Chi-square test, χ^2^ (*p*-value)
Total FACT-Cog Score	71 (29.3)	41 (33.6) (n = 122)	30 (25.0) (n = 120)	0.14
Mental acuity	71 (29.3)	43 (35.2) (n = 122)	28 (23.3) (n = 120)	0.042
Concentration	63 (26.1)	42 (35.0) (n = 122)	24 (17.6) (n = 119)	0.003
Memory	38 (15.8)	23 (18.9) (n = 122)	15 (12.6) (n = 119)	0.18
Verbal fluency	44 (18.2)	21 (17.2) (n = 122)	23 (19.2) (n = 120)	0.69
Functional Interference	45 (18.7)	28 (22.9) (n = 122)	19 (15.9) (n = 119)	0.08
Multitasking	63 (26.2)	35 (28.7) (n = 122)	28 (23.7) (n = 118)	0.38

^a^Data are presented as frequency (%) of patients.

### Mean plasma biomarker levels across study time points

Mean DHEAS levels in µM (expressed with ± standard-deviation) showed an overall reduction from baseline [T1: 2.80 ± 1.94, T2: 2.03 ± 1.56, T3: 1.96 ± 1.51, *p* < 0.001], with significant differences found between post-hoc comparisons at T2-T1 and T3-T1.

(Table 3). Mean DHEA levels in nM decreased from baseline [T1: 14.49 ± 10.92, T2: 13.37 ± 12.65, T3: 12.14 ± 10.95, *p* = 0.09] with significant difference found at T3-T1 (*p* < 0.001). In a subgroup of patients whose estradiol levels were available (n = 161), their mean levels in pM exhibited a decreasing trend followed by a small increase [T1: 191.90 ± 232.18, T2: 94.74 ± 116.64, T3: 98.21 ± 150.91, *p* < 0.001] with significant difference found at T2-T1 and T3-T1, although the mean estradiol level at the end of chemotherapy was still 2 times less than the baseline value.

**Table 3:** Mean plasma levels of biomarkers along hormonal pathway across the study time points.

Hormone	Mean (± SD) plasma levels			ANOVA	Post-hoc Test^a^		
	T1	T2	T3	*p*-value	T2-T1 *p*-value	T3-T2 *p*-value	T3-T1 p-value
**Pooled DHEAS**							
(µM) (n = 242)	2.80 ± 1.94	2.03 ± 1.56	1.96 ± 1.51	** < 0.001**	**< 0.001**	0.39	**< 0.001**
**Pooled DHEA**							
(nM) (n = 242)	14.49 ± 10.92	13.37 ± 12.65	12.14 ± 10.95	0.09	0.13	0.09	< 0.001
**Estradiol**							
(pM) (n = 161)	191.90 ± 232.18	94.74 ± 116.64	98.21 ± 150.91	** < 0.001**	** < 0.001**	0.78	**< 0.001**

^a^Bolded values indicate statistical significance with cut-off for Bonferroni-post hoc set at 0.0167.

### Difference in biomarker levels in relation to cognitive impairment

**Figure 2:**
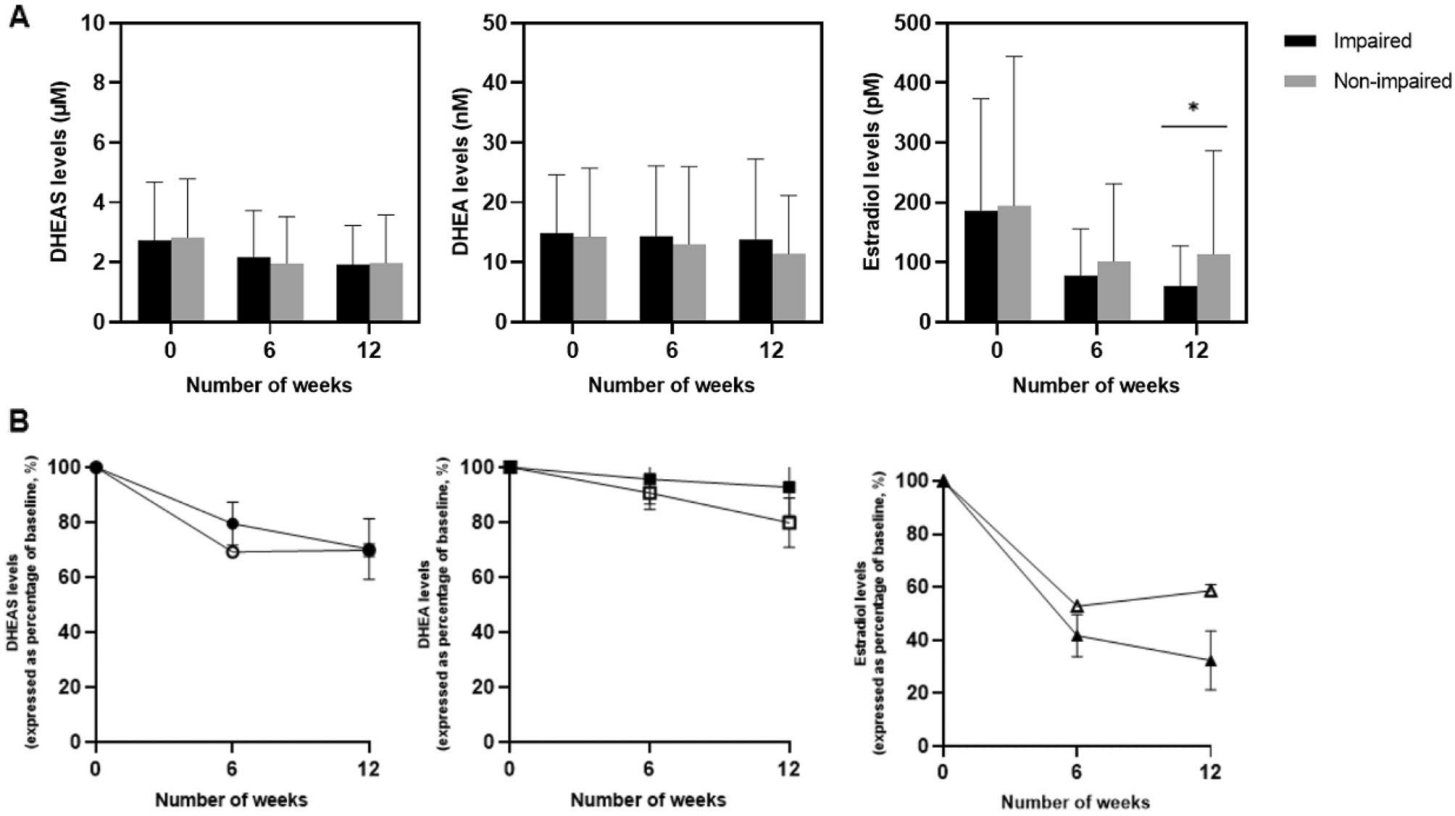
(**A**) Mean plasma DHEA(S) and estradiol levels (± standard deviation) stratified by overall impairment status. (**B**) Trajectory of plasma DHEA(S) and estradiol levels (expressed as percentage of baseline) stratified by overall impairment status.

The mean plasma DHEA(S) and estradiol biomarkers were plotted across the number of weeks, stratified by overall impairment status (Fig. 2A). The trajectory of plasma biomarkers levels was also expressed as a percentage of baseline in Fig. 2B. With regards to estradiol levels, it is noteworthy that mean levels were approximately twofold higher in the non-impaired group compared to impaired group at T3.

#### Differences in biomarker levels in relation to overall cognitive impairment status

The magnitude of change in biomarker levels between time points were compared between cognitive groups and adjusted for baseline levels of fatigue, anxiety, insomnia, menopausal status and age (Table 4).

**Table 4:** Change in mean biomarker levels from baseline stratified by impairment status at 6 and 12 weeks from baseline.

Time period: 6 weeks		Total global FACT-Cog score			PCI sub-scale			
Pooled Biomarker	Status	Change in mean levels (SD)	Adjusted difference in status variable β^a^ (95% CI)	*p*-value	Status	Change in mean levels (SD)	Adjusted difference in status variable β^b^ (95% CI)	*p*-value
DHEAS (μM)	Impaired (n = 71)	−0.56 (1.18)	**0.40** **(0.05, 0.74)**	**0.024**	Impaired (n = 83)	−0.54 (1.18)	**0.40** **(0.07, 0.74)**	**0.018**
	Non-impaired (n = 171)	−0.86 (1.20)			Non-impaired (n = 159)	−0.90 (1.20)		
DHEA (nM)	Impaired (n = 72)	−0.64 (11.57)	1.79 (−1.44, 5.01)	0.28	Impaired (n = 83)	−0.92 (13.62)	0.96 (−2.16, 4.08)	0.55
	Non-impaired (n = 171)	−1.33 (11.58)			Non-impaired (n = 159)	−1.24 (10.36)		
Estradiol (pM)	Impaired (n = 48)	−106.92 (133.86)	−1.99 (−53.93, 49.95)	0.94	Impaired (n = 56)	−115.23 (163.97)	−1.45 (−56.90, 54.00)	0.96
	Non-impaired (n = 113)	−92.18 (163.84)			Non-impaired (n = 106)	−89.78 (152.08)		

Time period: 12 weeks		Total global FACT-Cog score			PCI sub-scale			
Pooled Biomarker	Status	Change in mean levels (SD)	Adjusted difference in status variable β^a^ (95% CI)	*p*-value	Status	Change in mean levels (SD)	Adjusted difference in status variable β^b^ (95% CI)	*p*-value
DHEAS (µM)	Impaired (n = 71)	−0.81 (1.05)	0.06 (−0.28, 0.41)	0.72	Impaired (n = 83)	−0.89 (1.22)	0.16 (−0.17, 0.49)	0.33
	Non-impaired (n = 171)	−0.85 (1.21)			Non-impaired (n = 159)	−0.74 (1.03)		
DHEA (nM)	Impaired (n = 72)	−1.07 (13.50)	2.30 (−0.73, 5.32)	0.14	Impaired (n = 83)	−1.99 (11.93)	0.85 (−2.09, 3.78)	0.57
	Non-impaired (n = 171)	−2.88 (9.25)			Non-impaired (n = 159)	−2.54 (10.00)		
Estradiol (pM)	Impaired (n = 48)	−127.69 (152.19)	−37.42 (−101.21, 26.37)	0.25	Impaired (n = 56)	−131.40 (154.36)	−27.28 (−95.62, 41.05)	0.43
	Non-impaired (n = 113)	−81.02 (198.35)			Non-impaired (n = 106)	−81.68 (195.89)		

Βeta-coefficient represents the linear regression difference in mean biomarker levels between cognitive status for at least two time points (6 weeks relative to baseline and/or 12 weeks relative to baseline). Status is defined by reduction of more than 10.6 points in the total FACT-Cog scores ^a^ and by reduction of 8.8 points in the PCI-sub scale ^b^ respectively. Reference group refers to non-cognitively impaired patients. Adjusted model accounts for baseline levels of fatigue, anxiety, insomnia, menopausal status and age. Bolded values indicated statistical significance.

At 6 weeks (T2), cognitively impaired patients reported smaller extent of DHEAS level decline compared to non-impaired group. Comparing to baseline, the impaired group experienced a mean decrease of 0.56 µM in DHEAS levels compared to the 0.86 µM for the non-impaired group. This difference was found to be statistically significant in the adjusted model (β = 0.40, 95% CI (0.05, 0.74), *p* = 0.024). When impairment was defined based on the PCI-subscale scale of FACT-Cog, there was a similar observation. At 6 weeks, cognitively impaired patients reported significantly smaller extent of DHEAS level decline compared to non-impaired group in adjusted model (β = 0.40, 95% CI (0.07, 0.74), *p* = 0.018). At 12 weeks, the impaired group experienced a decline in DHEAS levels of 0.89 µM while there was a mean decrease of 0.74 µM in DHEAS for the non-impaired group. The β-coefficient for the difference in cognitive status variable was not significant for the adjusted model defined by total score (β = 0.06, 95% CI (−0.28, 0.41) *p* = 0.72) and PCI (β = 0.16, 95% CI (-0.17, 0.49), *p* = 0.33) at 12 weeks. The extent of change in DHEA and estradiol levels did not significantly differ between groups based on overall cognitive status at 6 weeks and 12 weeks.

### Impact of menopausal status on levels of biomarkers

The extent of change in biomarker levels was examined by menopausal status in Supplementary Table 2. The β-coefficient for menopausal status was significant in adjusted model for estradiol levels at 6 weeks. At 6 weeks, pre-menopausal group reported mean estradiol level decline of 151.70 pM compared to decline of 42.13 pM for post-menopausal group. The β-coefficient for the difference in estradiol levels between menopausal status was significant in the adjusted model (β = 114.25, 95% CI (39.70, 188.80), *p* = 0.003). The interaction between menopausal status and cognitive impairment status was not found to be significant for the change in mean levels of biomarkers.

## Discussion

In this longitudinal study, we observed how the hormonal profile of cancer patients had been affected after initiation of chemotherapy, with a significant reduction in estradiol and its precursors, DHEA(S) as patients underwent treatment. Our adjusted model has shown that patients reporting CRCI showed greater magnitude of decline in estradiol levels during and towards the end of chemotherapy, with a positive beta-coefficient representing greater extent in magnitude of biomarker level change from the non-impaired group (reference group) compared to impaired group. The decline in hormonal biomarkers could be attributed to the neurotoxicity of the drugs administrated, as alkylating agents and glucocorticoids that may induce adrenal insufficiency were reported to indirectly decrease DHEA(S) levels^23^. DHEA decline had also been linked to neuroinflammation in which the increased localised availability of DHEA may confer neuroprotective effect^24^. Noting just the decline in hormone levels may not be as informative since we should also consider how the magnitude of cognitive changes in relation to degree of estradiol decline. In a longitudinal study conducted among prostate cancer patients, the decline in estradiol was found to be relatively less than that of testosterone (which lies before estradiol in the biosynthesis pathway) during androgen deprivation^17^. With quantitative measurements of the biomarkers of interest, our study provided insights on the extent at which the reduction of biomarker levels contributes to the acute manifestation of CRCI symptoms.

Contrary to our hypothesis, we did not observe a significant interaction found between menopausal and cognitive impairment status. In another study, significant decrease in estradiol levels were shown in pre- and peri-menopausal breast cancer patients who reported decreased cognitive function within 2 to 3 weeks of completing adjuvant chemotherapy^34^. These observations support our hypothesis that lower estradiol levels (independent of menopausal status) would confer less neuroprotection on patients receiving acute treatment for cancer although an association between estradiol levels and cognitive complaints had not been observed in that study. Considering how age may be a contributing factor, younger women may be more likely to perceive more changes in cognitive function due to lifestyle challenges and experience a more abrupt decline in estradiol levels. This has substantiated our comparison of using age-adjusted magnitude of estradiol decline to investigate for its association with acute onset of CRCI.

When estradiol levels were put in perspective with its related precursors, there was interestingly a greater extent of decrease in overall DHEA(S) levels in the non-impaired group compared to CRCI patients. In particular, patients reporting acute CRCI showed significantly greater magnitude of decline in DHEAS levels than non-impaired group at 6 weeks from baseline (adjusted β of 0.40, *p*-value of 0.02). This trend ran contrary to our hypothesis as an individual would expect CRCI patients to have lower levels of both estradiol and DHEA(S) levels, given their purported neuroprotective effects and function that had been reported in literature. Since these biomarkers share a common conversion pathway, it may be biologically plausible that the direction of effect is inclined towards DHEA’s conversion to estradiol among patients without CRCI. If the assumption that circulating levels directly correlates with produced levels were to hold, a correspondingly greater reduction in DHEA levels would lead to more precursors being further converted to estradiol which may confer neuroprotection and buffer the impact on cognition from cancer treatment. This may provide a possible explanation for our findings as to why non-impaired patients did not experience cognitive impairment and showed an increase in estradiol levels towards the end of chemotherapy.

Conversely, there was a greater extent of decrease in estradiol relative to DHEA in patients reporting CRCI compared to non-impaired group. While we hypothesize there would be changes in the same direction of biomarker levels lying along the pathway, this may not necessarily hold true. In Okamoto et al., the percentile decrease in mean serum hormone levels of estradiol was found to be of about threefold more than that of DHEAS and DHEA in patients receiving luteinizing hormone-releasing hormone agonist^16^. Though of a different treatment modality, this shows that the impact of cancer treatment can affect the pathway to varying extent, with dysregulation of estradiol (in the upstream pathway) being affected to a greater extent than DHEA(S). In our instance, the impact may be inclined downstream for patients affected by CRCI (Fig. 1). There are two postulations to our observations: (i) there could be inefficient enzyme conversions such as of DHEA to androgens and subsequently to estradiol by aromatase; or DHEAS desulfonation to DHEA^31,32^, (ii) higher amount of DHEA(S) may have been produced to compensate for the reduction of estradiol. In addition, obesity is reported to be a patient-related factor that can influence measurement of estradiol levels^33,34^. No significant differences were observed by impairment status or the biomarker levels when the cohort is stratified by body mass index that is dichotomized to obesity or non-obesity. In comparison to DHEA, DHEAS levels may be indicative of the available reservoir of hormones as DHEAS reflects the predominant stable and circulating form but not necessarily the active one in conversion^18^.

Following our hypothesis that DHEA(S) levels are neuroprotective, the difference in precursors levels decline between the CRCI and non-impaired patients may be explained and attributed to other factors of metabolites and alternative sources of hormone production as well. As DHEA(S) can be converted into either androgenic and estrogenic metabolites, the net effect observed on cognition mediated by DHEA(S) may also need to account for the extent of conversion into either metabolites^35^. For instance, positive effect on memory which estrogens are known to enhance, may be offset by negative effects from androgens, and it is a combination of their varying levels that may exert different effects depending on the cognitive task assessed. It could be possible that in patients reporting CRCI, the impact of treatment on their androgen levels may be less than proportionate on estradiol levels. In addition, there are alternative sources for DHEAS production besides the adrenal cortex, ovarian, gonads and DHEAS may be produced even in neurons and glial cells, primarily astrocytes^20^. To be more precise on the physiological action, we may need to measure the levels in the form of non-protein bound that can cross the blood–brain barrier more readily or those that are produced de novo in the brain regions which can acts locally on the estrogen receptors to elicit cognitive effects. Nonetheless, measurement of the circulating levels in plasma may provide an adequate less-invasive measure of the endogenous levels, compared to sourcing from brain tissue or cerebrospinal fluid.

In another study, older women with subjective cognitive complaints were observed to have higher levels of DHEA(S) but the evidence supporting association between higher levels of plasma estrone and better performance on cognitive testing was modest, supporting that while the biomarkers share a common pathway, they may not necessarily be directly correlated^36^. An animal study conducted in female rhesus macaques failed to observe a corresponding increase in circulating and brain levels of estradiol after DHEA were being supplemented^37^. These results suggested that plasma levels of DHEA(S) may be insufficient to serve as effective precursors for estrogen synthesis in the brain. While an association between the magnitude of decline in circulating DHEAS levels was found with acute CRCI in our study, their levels may not be representative of the available precursors levels that can get converted into estradiol in the brain. Another study reported that increased levels of estradiol could downregulate estrogen synthesis from DHEA and de novo DHEA synthesis in the brain^38^. One should also consider age-related decline of DHEA(S) reserves. Given the multitude of factors that could potentially influence the measured hormone levels, more experimental research on the biochemical energetics and specific component that had been implicated in the hormonal milieu is needed to improve our mechanistic understanding of how these biomarkers may influence CRCI.

The strength of this study lies in analysing the temporal trends between biomarkers related to hormonal pathway and the cognitive trajectory. There were also standardized study time points for the blood samples which coincided with the different phases of each patient (before, and with varying duration of exposure to chemotherapy). Nonetheless, we acknowledged some limitations in our study: The baseline sample collection need not necessarily coincide with the menstrual cycle of the assessed patient. In addition, cognitive status was based on a self-reported tool instead of a neuropsychological battery that could provide objective measure. Given the observational nature of our data, there could be unmeasured and residual confounding inherent despite our attempts to control for them through statistical analysis. Furthermore, there may be potential for misclassification bias as some of the clinical variables such as menopausal status are self-reported, and the degree of variation for the physiological range of biomarkers was found to be wide between cognitive categories. A longer duration of follow-up ranging from 3 to 5 years (typical of the duration of adjuvant endocrine therapy) could be useful to investigate the long-term impact of hormone level decrease on cognitive trajectory. In fact, chemotherapy-induced menopause and CRCI may be reversible, and it would be interesting to see if cognitive impairment of any kind would persist after the resumption of cycles and normalization of biomarkers.

To the best of our knowledge, this is one of the first study to report an association between magnitude of change in biomarker levels with self-reported CRCI. However, our study was not designed to isolate the multiple overlapping pathways and physiological factors involved. In that regard, future research on the mechanistic understanding of how suppression of biomarker levels contribute to CRCI may be warranted. This may allow researchers to develop targeted intervention-a potential application in which drugs targeting HPA axis-related hormones such as DHEA or sex steroids had shown promising cognitive-enhancing properties in patients with major mood disorders and schizophrenia^39^.

## Conclusion

In conclusion, there was a significant association reported between decline in DHEAS levels and acute onset of CRCI. During adjuvant chemotherapy at 6 weeks from baseline, we observed smaller magnitude of decline in DHEA(S) levels in patients reporting CRCI compared to non-impaired patients. On the contrary, we observed a greater magnitude of decline of estradiol levels among patients with cognitive complaints. There needs to be a further understanding of the biological pathway from which DHEAS may implicate and corelate with cognitive dysfunction. Our findings may help to identify sub-group of breast cancer patients who may be at higher risk of developing CRCI and require early rehabilitation to prevent cognitive side effects.

## Supplementary Information

Supplementary Information 1.

Supplementary Information 2.

## Data Availability

The datasets generated during and/or analysed during the current study are available from the corresponding author on reasonable request.
