# Supplementary figures and images for: Longitudinal evaluation of dehydroepiandrosterone (DHEA), its sulfated form and estradiol with cancer-related cognitive impairment in early-stage breast cancer patients receiving chemotherapy
Source: Sci Rep. 2022 Oct 3;12:16552. doi: 10.1038/s41598-022-20420-3 (PMC9529889; doi:10.1038/s41598-022-20420-3)

**Supplementary Figure 1: Study flow diagram**

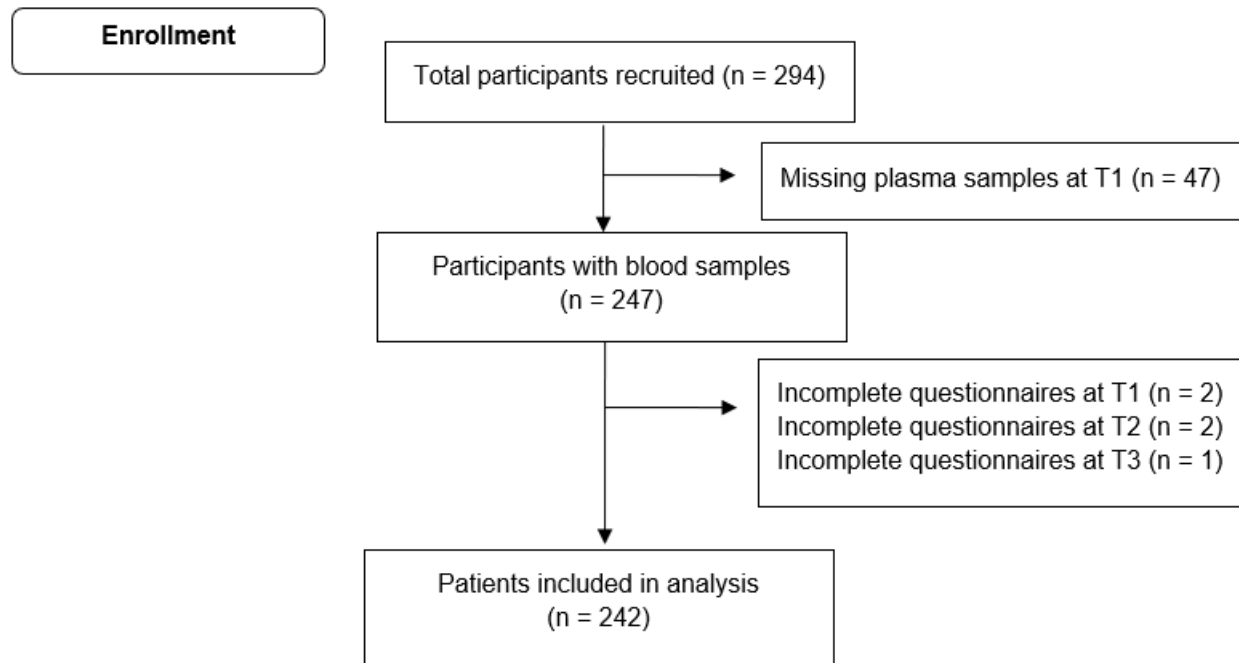

Supplement: Supplementary file 1 — Supplementary Information 1. [file 41598_2022_20420_MOESM1_ESM.pdf]
