# Supplementary material for: Longitudinal evaluation of dehydroepiandrosterone (DHEA), its sulfated form and estradiol with cancer-related cognitive impairment in early-stage breast cancer patients receiving chemotherapy
Source: Sci Rep. 2022 Oct 3;12:16552. doi: 10.1038/s41598-022-20420-3 (PMC9529889; doi:10.1038/s41598-022-20420-3)
Supplement: Supplementary file 2 — Supplementary Information 2. [file 41598_2022_20420_MOESM2_ESM.docx]

**Supplementary Table 1:** Comparison of demographic and clinical characteristics of patients included and excluded from analysis

| **Demographic information** | | **Included patients**  **(n = 242)** | **Excluded patients**  **(n = 52)** | **p-value** |
| --- | --- | --- | --- | --- |
| **Age (years)** | | 50.8 ± 9.2 | 52.5 ± 8.6 | 0.23 |
| **Years of education** | | 11.2 ± 3.4 | 10.8 ± 3.1 | 0.48 |
| **Ethnicity** | Chinese | 203 (83.9) | 34 (65.4) | 0.016 |
|  | Malay | 23 (9.5) | 9 (17.3) |  |
|  | Indian | 9 (3.7) | 6 (11.5) |  |
|  | Others | 7 (2.9) | 3 (5.8) |  |
| **Breast Cancer Stage** | I | 30 (12.4) | 14 (27.5) | 0.018 |
|  | II | 153 (63.2) | 30 (58.8) |  |
|  | III | 58 (24.0) | 7 (13.7) |  |
| **ECOG Status** | 0 | 229 (94.6) | 52 (100.0) | 0.087 |
|  | 1 | 13 (5.4) | 0 (0.0) |  |
| **Chemotherapy Regimen** | Anthracycline-based | 166 (68.6) | 23 (44.2) | <0.001 |
|  | Taxane-based | 76 (31.4) | 29 (55.8) |  |
| **Menopausal status** | Pre-menopausal | 122 (50.4) | 24 (46.2) | 0.58 |
|  | Post-menopausal | 120 (49.6) | 28 (53.8) |  |

^a^Data are presented as mean ± standard deviation for continuous variables and frequency (%) for categorical variables.

**Supplementary Table 2**: Change in mean biomarker levels from baseline stratified by menopausal status (without adjusting for baseline levels)

|  |  | **6 weeks** | | | | | **12 weeks** | | | | |
| --- | --- | --- | --- | --- | --- | --- | --- | --- | --- | --- | --- |
| **Pooled Biomarker** | **Status** | **Change in mean levels (SD)** | **Difference in status variable β^a^ (95% CI)** | **p-value** | **Adjusted β^b^ (95% CI)** | **p-value** | **Change in levels (SD)** | **Difference in status variable β^a^ (95% CI)** | **p-value** | **Adjusted β^b^ (95% CI)** | **p-value** |
| **DHEAS (µM)** | Pre-menopausal (n=122) | -0.98 (1.32) | 0.41 (0.11, 0.71) | **0.007** | 0.23 (-0.25, 0.71) | 0.35 | -0.88 (1.29) | 0.08 (-0.21, 0.37) | 0.59 | -0.09 (-0.56, 0.37) | 0.69 |
|  | Post-menopausal (n=120) | -0.57 (1.03) |  |  |  |  | -0.80 (1.01) |  |  | -0.26 (-0.79, 0.27) | 0.33 |
| Adjusted β for CRCI^c^  (95% CI) |  |  |  |  | 0.41 (-0.06, 0.88) | 0.086 |  |  |  | -0.15 (-0.62, 0.31) | 0.52 |
| Interaction term^d^ |  |  |  |  | -0.027 (-0.73, 0.67) | 0.94 |  |  |  | 0.48 (-0.21, 1.17) | 0.17 |
| **DHEA (nM)** | Pre-menopausal (n=122) | -0.85 (12.01) | -0.55 (-3.49, 2.37) | 0.71 | -3.17 (-7.60, 1.24) | 0.16 | -1.08 (12.39) | -2.56 (-5.25, 0.13) | 0.062 | -2.28 (-6.45, 1.87) | 0.28 |
|  | Post-menopausal (n=120) | -1.41 (11.12) |  |  |  |  | -3.64 (8.45) |  |  |  |  |
| Adjusted β for CRCI^c^  (95% CI) |  |  |  |  | 3.16 (-1.20, 7.52) | 0.15 |  |  |  | -0.99 (-5.69, 3.71) | 0.68 |
| Interaction term^d^ |  |  |  |  | -3.05 (-9.55, 3.45) | 0.36 |  |  |  | -3.10 (-9.20, 2.99) | 0.32 |
| **Estradiol (pM)** | Pre-menopausal (n=81) | -151.70 (183.18) | 109.57 (64.23, 154.91) | < 0.001 | **114.25 (39.70, 188.80)** | **0.003** | -157.62 (215.00) | **123.29 (67.94, 178.66)** | **< 0.001** | 77.09 (-14.57, 168.76) | 0.10 |
|  | Post-menopausal (n=84) | -42.13 (94.98) |  |  |  |  | -34.33 (129.49) |  |  |  |  |
| Adjusted β for CRCI^c^  (95% CI) |  |  |  |  | -11.77 (-84.57, 61.03) | 0.75 |  |  |  | -64.27 (-154.15, 25.61) | 0.16 |
| Interaction term^d^ |  |  |  |  | 20.06 (-84.24, 124.35) | 0.70 |  |  |  | 54.24 (-73.54, 182.01) | 0.40 |

^a^Beta-coefficient represents the linear regression difference in mean biomarker levels between menopausal status. Reference group refers to pre-menopausal patients. ^b^Adjusted model accounts for baseline levels of fatigue, anxiety, insomnia and age. ^c^Beta-coefficient for cognitive impairment status in model testing for interaction ^d^Interaction term refers to beta-coefficient of interaction term between menopausal status and cognitive status in adjusted model with main effects.
